# Transposable elements as a possible missing link between genetic predisposition and environmental triggers of autoimmune disorders: insights from type 1 diabetes, systemic lupus erythematosus and rheumatoid arthritis

**DOI:** 10.3389/fimmu.2026.1774863

**Published:** 2026-04-30

**Authors:** Karolina Mužina, Ana Markež Vrhovšek, Barbara Jenko Bizjan, Neja Šamec, Jernej Kovač, Klemen Dovč, Tadej Battelino, Marko Pokorn

**Affiliations:** 1 University Children’s Hospital, University Medical Centre Ljubljana, Ljubljana, Slovenia; 2 Faculty of Medicine, University of Ljubljana, Ljubljana, Slovenia

**Keywords:** autoimmune disease, autoimmunity, epigenetic, methylation, RNA-seq, sequencing, transposable elements

## Abstract

Transposable elements (TEs) make up almost half of the human genome and are among its most densely methylated regions. Their epigenetic silencing is crucial for genomic stability and immune homeostasis, and accumulating evidence indicates that dysregulated TE methylation and expression contribute to autoimmune disease pathogenesis. Hypomethylation of selected TE families can permit transcriptional reactivation, production of immunostimulatory nucleic acids and peptides, and engagement of pattern-recognition receptors, thereby driving type I interferon (IFN-I) signaling through “viral mimicry”–like mechanisms. In parallel, TE-derived enhancers, promoters and exons reshape gene regulatory networks at immune loci. In this narrative review, we synthesize current knowledge on TE methylation and expression in autoimmunity, with a focus on type 1 diabetes (T1D), systemic lupus erythematosus (SLE) and rheumatoid arthritis (RA). We first outline TE biology and the principal mechanisms of epigenetic silencing, then summarise methodological advances for TE methylation and expression profiling, including long-read sequencing and TE-aware RNA-seq pipelines. We next dissect disease-specific evidence: longitudinal epigenomic studies in T1D showing preclinical DNA methylation changes and altered Alu/LINE-1 patterns, together with HERV-H/W upregulation at onset, SLE studies demonstrating LINE-1 hypomethylation in neutrophils and cell-type–specific TE overexpression that tracks with IFN signatures and nucleic acid sensor pathways, and RA studies linking global and LINE-1 methylation to methotrexate response when integrated with serostatus. Across conditions, TE methylation behaves more like a relatively stable disease-associated trait than a simple activity marker and exhibits clear disease- and cell-type-specific signatures rather than global hypomethylation. We conclude that TEs are not passive genomic relics but epigenetically regulated elements that can act as endogenous sources of immunostimulatory nucleic acids, neoantigens and regulatory sequences, providing a mechanistic bridge between genetic susceptibility and environmental triggers in autoimmunity. Consequently, selective dysregulation of TE methylation and expression offers both an explanatory framework for interferon-driven autoimmunity and a promising, currently underused layer for biomarker development and therapeutic targeting.

## Graphical Abstract

**Figure:**
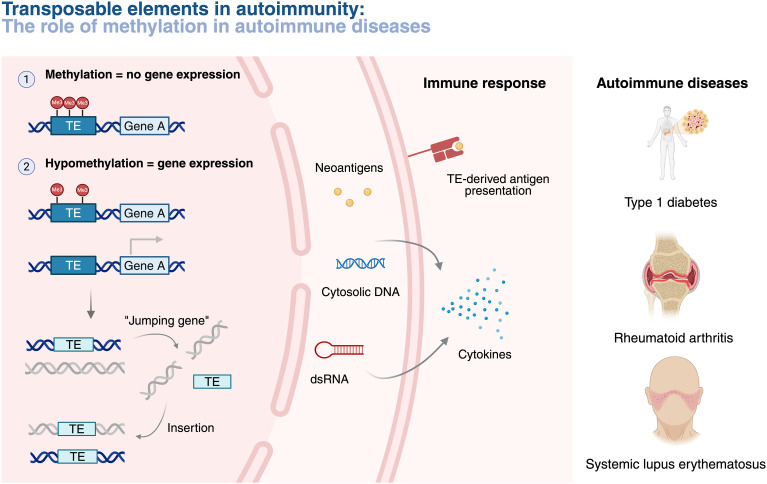

## 1 Introduction

Epigenetics encompass structural and biochemical modifications of chromatin that occur without altering the underlying primary DNA sequence (1). These modifications play critical roles in development, cellular differentiation, and disease pathogenesis (2, 3). Four primary mechanisms regulate gene expression: DNA methylation, histone modification, chromatin remodeling, and non-coding RNAs (ncRNAs) (3). DNA methylation plays a crucial role in regulating gene expression and maintaining genomic stability (4, 5). In mammalian genomes a main type of DNA methylation encompasses the methylation at the carbon-5 position of cytosine (resulting in 5-methylcytosine, or 5mC). This review will focus on DNA methylation referring to the covalent addition of a methyl group to the 5-position of cytosine residues, occurring predominantly within CpG dinucleotides (5). In most somatic cells and tissues, approximately 60–80% of more than 32 million CpG sites in the human genome are methylated (6, 7).

**Figure 1:**
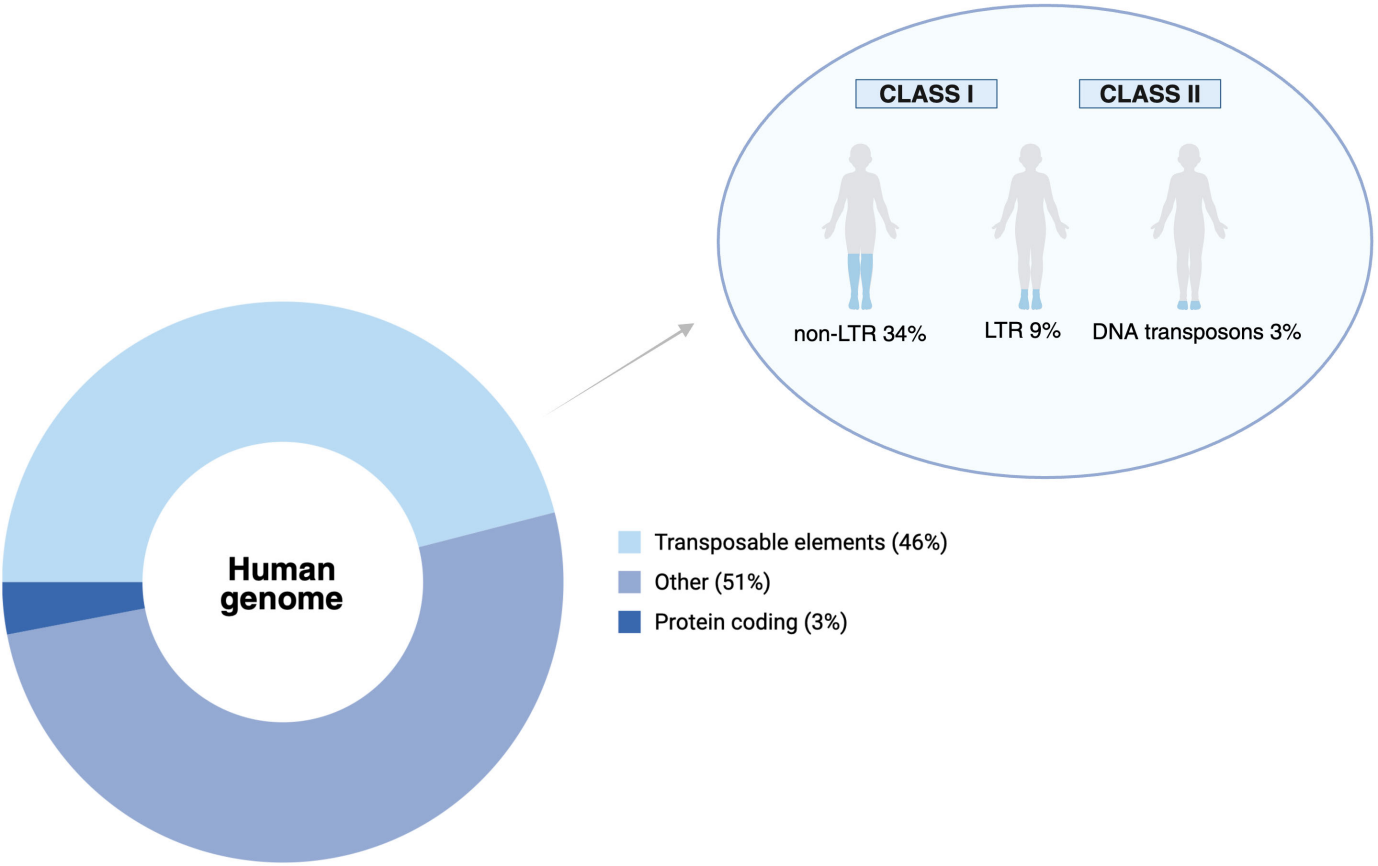
Composition of the human genome and relative contribution of transposable elements.

Transposable elements (TEs), which make approximately 46% of the human genome (**Figure 1**), were once considered genomic parasites. Now they are recognized as significant players in shaping gene regulation and immune responses (8, 9). Also referred to as “jumping genes”, TEs are mobile DNA sequences capable of inserting themselves into various locations throughout the genome and are often silenced epigenetically (10, 11).

Donut chart depicting the approximate sequence composition of the human genome, with protein-coding regions representing only a small fraction (~3%), compared with transposable elements (TEs; ~46%) and other non-coding sequences (~51%), including introns, regulatory regions and low-complexity repeats. The inset illustrates the breakdown of the TE compartment into its major groups: non-LTR retrotransposons (~34% of the genome; mainly LINEs and SINEs), LTR retrotransposons (~9%; including HERVs) and DNA transposons (~3%). The figure emphasizes that TEs constitute the largest defined functional category of genomic sequence and are prime targets of epigenetic silencing, while at the same time providing a rich source of regulatory elements whose dysregulation can influence immune homeostasis and autoimmunity, as discussed in this review.

This review brings together current insights into how TE activity and DNA methylation contribute to autoimmune disease mechanisms, highlighting their interconnected roles and exploring their promise as targets for future therapies. Understanding the interplay between TEs and their DNA methylation is crucial for understanding the epigenetic factors involved in autoimmunity.

## 2 Methods

For this literature review, we conducted a comprehensive search for relevant articles on PubMed, Google Scholar, and ScienceDirect. The search was performed between May and November 2025, focusing on peer-reviewed studies related to methylation of TEs elements and autoimmunity. Key search terms included “methylation”, “transposable elements”, “autoimmune disease”, “epigenetic”, “hypomethylation”, “autoimmunity”, “RNA-seq” and “sequencing”. No geographical restrictions were applied and studies from all countries and written in English were considered. The citations from all relevant studies and reviews were reviewed to identify additional articles.

## 3 Transposable elements and epigenetic silencing

### 3.1 What are transposable elements

**Figure 2:**
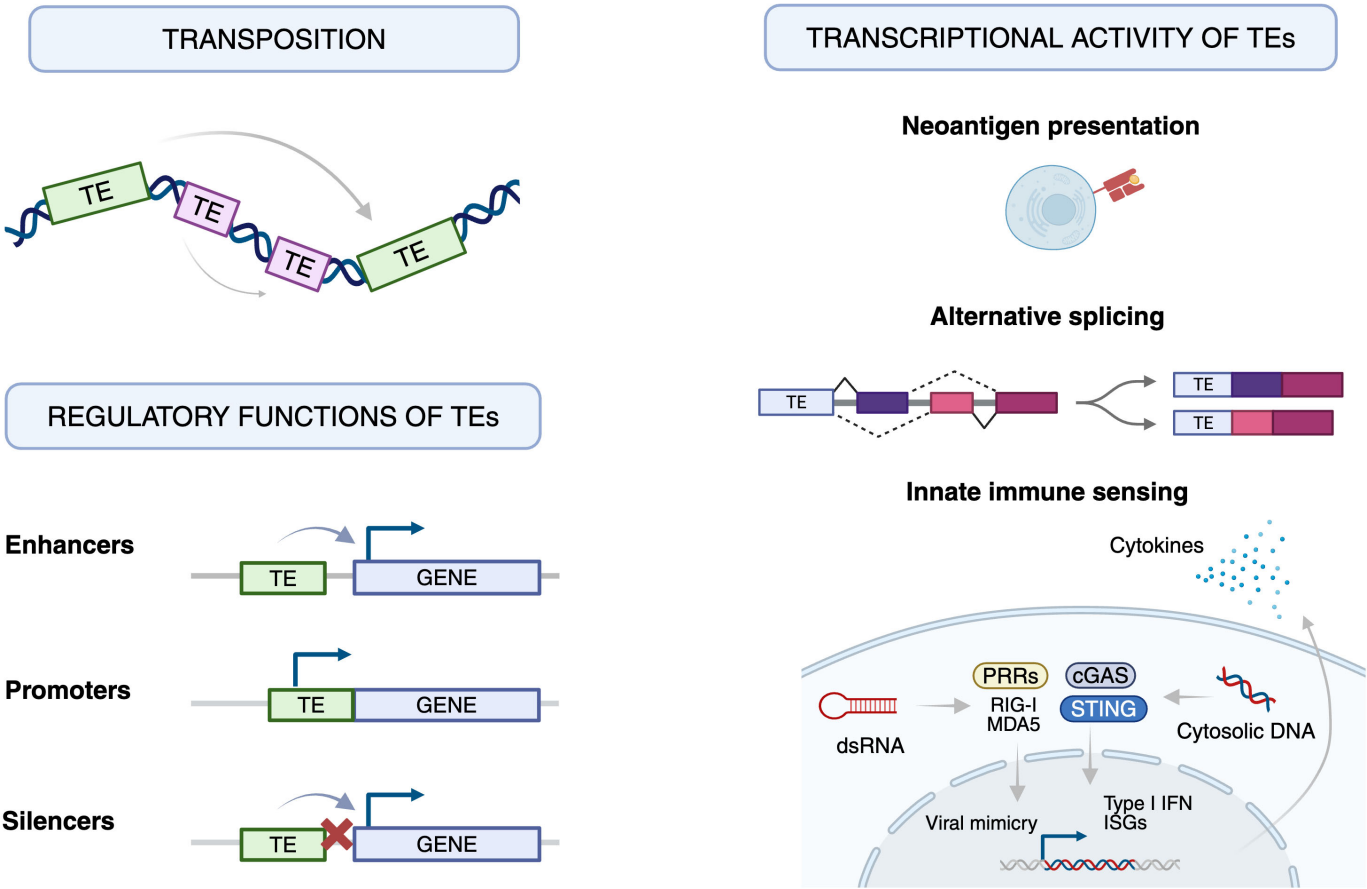
Major classes of transposable elements and their canonical modes of mobilization.

TEs are generally classified into two major types: class I (retrotransposons) and class II (DNA transposons) (**Figure 2**). Class I, or retrotransposons, retrotranspose via a *copy-and-paste* mechanism using an RNA intermediate (12). This RNA is reverse transcribed into DNA by a reverse transcriptase and then inserted into a new location in the genome, leading to an increase in copy number (13, 14). Retrotransposons are the most abundant class of TEs in the human genome. This group is further divided into two subclasses: long terminal repeat (LTR) retrotransposons and non-LTR retrotransposons. These include long interspersed nuclear elements (LINEs) – with LINE-1, which is approximately 6 kilobase pairs (kbp) in length, being the most prominent example, and short interspersed nuclear elements (SINEs) like Alu elements, which are around 300 base pairs (bp) long. LTR elements include human endogenous retroviruses (HERVs), typically spanning between 7 and 10 kilobases (kb) in size (9, 12, 15–17). Class II DNA transposons mobilize via a *cut-and-paste* mechanism involving direct DNA transposition without an RNA intermediate. These are further divided into subclass I (e.g. TIR, Crypton) and subclass II (e.g. Helitrons, Mavericks) (10, 12).

Transposable elements are broadly divided into Class I retrotransposons and Class II DNA transposons. Left, Class I retrotransposons transpose via an RNA intermediate in a “copy-and-paste” mechanism in which the original element remains at the donor site while a new copy is inserted elsewhere in the genome. This class comprises LTR retrotransposons (such as human endogenous retroviruses, HERVs) and non-LTR retrotransposons, including autonomous LINEs (e.g. LINE-1) and non-autonomous SINEs (e.g. Alu). Right, Class II DNA transposons move by a “cut-and-paste” mechanism, whereby the element is excised from its original position and reinserted at a new genomic location. These are subdivided into subclass I elements (such as TIR transposons and Cryptons) and subclass II non-TIR transposons. Although most human TEs are now immobile, their distinct origin and mobilization mechanisms have left a large, diverse reservoir of sequences that are differentially targeted by epigenetic silencing and can be co-opted as regulatory elements or, when dysregulated, contribute to genome instability and aberrant immune activation discussed in this review.

### 3.2 Mechanisms regulating transposable elements

Activity and regulation of TEs play a major role in gene expression and genome stability. TE regulation is controlled by a combination of epigenetic mechanisms, protein repressors, and RNA-based pathways, which collectively determine how TEs influence gene expression both locally and across the genome (18–20). DNA methylation and histone modifications are key epigenetic mechanisms that regulate gene expression, particularly in silencing TEs, which pose a threat to genome integrity (21). The maintenance and deposition of 5mC are controlled by DNA methyltransferases (DNMTs), such as DNMT1 which maintains methylation patterns, and DNMT3A, and DNMT3B which are required for new methylation patterns, and all of them are essential during development and in germ cells (21). Complementary to DNA methylation, histone modifications, especially histone H3 lysine 9 trimethylation (H3K9me3), play an important role in forming repressive chromatin. Proteins such as KRAB-ZFPs recruit cofactors like KAP1 and SETDB1, which promote H3K9 methylation at TE loci, reinforcing silencing (21, 22). This system is active in both embryonic and adult tissues and modulates gene networks by controlling TEs (23). Recent evidence indicates that these two silencing mechanisms, DNA methylation and H3K9me3, are not equally important, with DNA methylation playing a dominant role in TE repression. Wang et al. demonstrated that knockout of DNMT1 causes IAP element derepression even when H3K9me3 levels are preserved, whereas SETDB1 loss reduces both H3K9me3 and DNA methylation marks (24). These findings suggest that H3K9me3 functions partly by maintaining DNA methylation at TE loci rather than independently silencing TEs, establishing DNA methylation as the primary determinant of TE silencing in differentiated cells (24). Recent analyses on human samples show that TEs frequently overlap with epigenomic features, with the extent of overlap varying by histone mark. Notably, the repressive mark H3K9me3 exhibits the highest TE overlap (78.3%), particularly enriched at L1 elements across all tissues, suggesting that H3K9me3 specifically targets L1 for silencing (25). Beyond transcriptional repression, post-transcriptional and post-translational mechanisms provide additional layers of TE control. A well-characterized example is MOV10, an RNA helicase that restricts LINE-1 (L1) retrotransposition primarily through post-transcriptional mechanisms, including binding of L1 RNA, interference with ribonucleoprotein (RNP) complex formation, and inhibition of reverse transcription. MOV10 itself is a subject of post-translational regulation via ubiquitination. Similar post-translationally regulated restriction factors include APOBEC3 enzymes and SAMHD1, whose activity and stability are controlled by ubiquitination and phosphorylation, respectively, as well as TRIM28 (KAP1), a chromatin-associated repressor regulated by SUMOylation (26, 27). A well-known process in the germline involves extensive interactions between TEs and various non-coding RNAs (18). PIWI-interacting RNAs (piRNAs) have been the most extensively studied for their role in regulating TEs. PiRNAs, 21–35 nucleotides in length, can silence gene expression in multiple ways, including transcriptional repression and epigenetic modifications that alter DNA accessibility for transcription-related proteins (19, 28, 29). These piRNAs guide epigenetic silencing by inducing chromatin modifications that block transcription or by degrading TE transcripts (30). In addition to piRNAs, double-stranded RNA derived from bidirectional TE transcription can be processed by Dicer into small interfering RNAs (siRNAs). These siRNAs are incorporated into the RNA-induced silencing complex (RISC), which mediates post-transcriptional cleavage of complementary TE transcripts. However, the siRNA pathway plays a more limited role in TE control in mammals compared to germline piRNA silencing (18, 30).

## 4 Hypomethylation of transposable elements

In mammalian genomes, TEs constitute some of the most densely methylated regions, with CpG sites within LINEs, SINEs and endogenous retroviral sequences typically carrying higher levels of 5-methylcytosine than neighboring unique sequences, reflecting strong evolutionary pressure to keep these potentially mobile elements stably repressed (13, 21, 31). DNA methylation acts as a constitutive silencer of TEs, maintaining genomic stability by keeping these repetitive elements transcriptionally inactive (18). TEs are generally repressed in somatic tissues, but they can become transcriptionally active during early embryonic development or under conditions of stress such as infection, inflammation, or oxidative damage (10). Ten-eleven translocation (TET) enzymes convert 5mC into oxidized forms such as 5-hydroxymethylcytosine (5hmC), 5-formylcytosine (5fC), and 5-carboxylcytosine (5caC) as part of an active, replication-independent DNA demethylation process (32). Beyond their role in removing DNA methylation, TET enzymes are increasingly recognized as key regulators of TE activity, either through demethylation or the functional effects of the modified cytosines they produce (21). Loss of methylation at TEs can lead to their reactivation, which may trigger immune responses (33).

The process by which TE hypomethylation triggers innate immune responses follows a sequential cascade of molecular events that culminates in activation of antiviral defense pathways. When DNA methylation is lost at TE loci, chromatin becomes accessible and transcription factors can bind to TE promoters, initiating bidirectional transcription (34). This produces complementary sense and antisense RNA transcripts that hybridize to form double-stranded RNA (dsRNA) a molecular pattern typically associated with viral infection (35, 36). Concurrently, LINE-1 elements that encode functional reverse transcriptase machinery can generate cytoplasmic DNA through reverse transcription of their own transcripts, producing cDNA and RNA-DNA hybrid intermediates (37). Tunbak et al. (38) further suggest that LINE-1 may also form dsRNA as a result of antisense transcription in their model system; however, direct experimental evidence for long dsRNA formation from LINE-1 transcripts is still lacking. This reverse transcriptase activity is not strictly limited to LINE-1 RNA itself, as SINE RNAs can serve as templates in trans, leading to the accumulation of cytoplasmic SINE-derived cDNA species and RNA–DNA hybrid intermediates (39).

The innate immune system employs three major pattern recognition receptor (PRR) systems to detect these TE-derived nucleic acids. The cGAS-STING pathway serves as the primary sensor for cytoplasmic DNA. Cyclic GMP-AMP synthase (cGAS) detects LINE-1-derived cDNA and reverse transcription intermediates, catalyzing production of the second messenger 2’3’-cGAMP, which activates stimulator of interferon genes (STING) (40, 41). STING subsequently activates TANK-binding kinase 1 (TBK1), leading to phosphorylation of interferon regulatory factor 3 (IRF3) and induction of type I interferon genes (37, 42). The RIG-I/MDA5-MAVS pathway detects dsRNA formed through bidirectional ERV and LINE-1 transcription. Melanoma differentiation–associated protein 5 (MDA5, encoded by IFIH1) is a principal cytosolic sensor of endogenous double-stranded RNA. While ERV-derived dsRNA has been shown to activate MDA5, a major physiological source of endogenous MDA5 ligands in human cells arises from inverted-repeat Alu elements (IRAlus) located within transcripts, which can form long, imperfect dsRNA structures. These IRAlu duplexes are extensively edited by ADAR1, and this editing reduces dsRNA stability, thereby limiting activation of MDA5 and preventing downstream inflammatory signalling and ZBP1-mediated cell death. In addition to Alu-derived dsRNA, LINE-1 transcripts may also generate immunostimulatory RNA species, and both MDA5 and RIG-I have been implicated in sensing LINE-1–associated dsRNA. However, direct evidence demonstrating binding of LINE-1–derived dsRNA by MDA5 remains limited (35, 36, 38, 43–45). MDA5appears to be the dominant sensor for ERV-derived dsRNA, while both MDA5 and retinoic acid-inducible gene I (RIG-I) can recognize LINE-1-derived dsRNA (35, 36, 38). Upon dsRNA binding, these receptors signal through the mitochondrial adaptor protein MAVS to activate IRF3, IRF7, and NF-κB, inducing interferons and inflammatory cytokines (46, 47). A third layer involves Toll-like receptors (TLRs): TLR3 in endosomes detects dsRNA, TLR7/8 recognize single-stranded RNA, and TLR9 detects unmethylated CpG DNA, a signature enriched in normally methylated TE sequences (48). This phenomenon, termed “viral mimicry,” was conclusively demonstrated in two landmark 2015 studies. Chiappinelli et al. showed that DNA methyltransferase (DNMT) inhibitor treatment in ovarian cancer cells reactivates ERVs, producing cytosolic dsRNA that activates the MDA5-MAVS-IRF7 pathway and induces type I interferon responses (49). Simultaneously, Roulois et al. demonstrated that low dose decitabine (a DNMT inhibitor) activates the same pathway in colorectal cancer cells, with genetic disruption of MDA5 or MAVS abolishing the interferon response (50). Importantly, the threshold between physiological and pathological TE expression appears context-dependent: controlled TE activation serves beneficial roles during development and immune homeostasis, while dysregulated chronic activation drives autoimmune pathology (51). In autoimmune diseases such as systemic lupus erythematosus and Aicardi-Goutières syndrome, constitutive TE-driven interferon production creates a state of chronic immune activation that contributes to tissue damage and disease progression (43, 52). Complementary evidence from non-malignant systems shows that TE activation can drive viral mimicry and sterile immune activation. De Cecco et al. reported that during late cellular senescence, LINE-1 (L1) expression increases in parallel with strong induction of type I interferons and interferon-stimulated genes, distinct from the early DNA damage response and classical senescence-associated secretory phenotype (SASP). They also showed that inhibiting reverse transcription with lamivudine (3TC) reduces this interferon response, indicating that retrotransposon replication intermediates—rather than transcription alone—trigger immune activation (53). Extending this mechanism to DNA sensing, Simon et al. showed that impaired chromatin regulation leads to accumulation of cytoplasmic L1 cDNA, which is sensed by cGAS and induces type I interferon production. This response is reduced by cGAS depletion or reverse transcriptase inhibition. They further demonstrated that aged mouse tissues display increased L1 activity and interferon signaling, both attenuated by reverse transcriptase inhibitors, linking endogenous retrotransposons to sterile inflammation *in vivo* (54).

While there is strong evidence linking abnormal DNA methylation to autoimmune disease onset, direct research specifically connecting TE methylation changes to autoimmune diseases is limited (55, 56). Early evidence linking DNA methylation to autoimmunity emerged from studies on T lymphocytes. Experiments showed that treating CD4+ T cells with the methylation inhibitor 5-azacytidine (5-azaC) triggered autoreactivity; the cells responded to self-major histocompatibility complex (MHC) class II molecules even without the relevant antigen (57).

Xie et al. (31) mapped tissue-specific TE hypomethylation across human tissues, revealing non-random patterns of demethylation. Although the location of these hypomethylated TEs near genes seems random overall, the functions of the nearby genes were highly enriched for tissue-specific biological roles. For example, two endogenous retroviruses (ERV)-LTR12 and LTR77-were hypomethylated in immune cells. Genes located near these TEs were enriched in immune functions, such as antigen processing and presentation via MHC class II and in the redox processes, which are important for T cell function (31). These findings indicate that tissue-specific hypomethylation of TEs can play a regulatory role, possibly by acting as enhancers or promoters for genes needed in that tissue.

## 5 Methods for DNA methylation and transposable element expression analysis

DNA methylation is usually detected using bisulfite-based methods, which still represent a gold-standard technology. Sodium bisulfite treatment converts unmethylated cytosines to uracils while leaving methylated cytosines unchanged, thereby enabling single-base resolution analysis of methylation patterns (13, 58). Whole-genome bisulfite sequencing (WGBS) provides comprehensive methylation profiles, while targeted bisulfite sequencing can focus on repetitive elements like LINE-1 and Alu (59, 60). The main limitation of bisulfite sequencing is harsh chemical treatment that causes significant DNA degradation, yielding only short fragments for analysis. Other drawbacks include reduced specificity, limited sequence diversity due to the short fragments generated, and challenges in haplotyping, since shorter reads are less likely to capture single-nucleotide polymorphisms (SNPs) (13). Recent advances in sequencing and bioinformatics have introduced novel strategies for interrogating DNA methylation and TE expression, enabling more accurate and comprehensive analyses than traditional methods (61). These innovations are valuable in the study of autoimmune diseases, where epigenetic changes and aberrant TE activity shape immune dysregulation (62).

### 5.1 DNA methylation detection: third-generation sequencing

Long-read sequencing platforms, including Oxford Nanopore Technologies (ONT) and PacBio single-molecule real-time (SMRT) sequencing allow direct detection of DNA modifications such as 5-methylcytosine (5mC) and 5-hydroxymethylcytosine (5hmC) without chemical conversion. These methods are particularly advantageous for repetitive regions like LINEs, SINEs, and endogenous retroviruses, which are often poorly covered by short-read approaches due to multi-mapping. Unlike short-read sequencing, long reads can cover entire TEs, resolving their full sequences and precise genomic location, even in highly repetitive regions like TEs. ONT enables long-read sequencing by passing native DNA strands through nanopores - tiny biological pores embedded in a membrane – while measuring changes in electrical current. These current disruptions are interpreted to identify the sequence of nucleotides, including epigenetic modifications. Resolving individual TE copies and their insertions is essential for understanding their impact on genome evolution, gene regulation, and disease (13, 63, 64).

### 5.2 Transposable element expression detection via RNA-seq

TEs exist in hundreds to millions of copies across the human genome. Many of these copies are highly similar in sequence. Retrotransposons (e.g. LINEs, SINEs, LTR) are transcribed into RNA as part of their life cycle. However, DNA transposons can also be transcribed, but their RNAs are generally not intermediates for transposition. Poly(A) tails are common in retrotransposon RNAs, such as LINE and SINE RNAs, as well as some LTR retrotransposons. This is important because most RNA-seq protocols use oligo(dT) primers to capture and sequence polyadenylated RNAs. Since many TE transcripts have poly(A) tails, these methods efficiently enrich for and detect their expression. When performing RNA-seq or single-cell RNA-seq (scRNA-seq), sequencing reads from TE transcripts often map to multiple locations, making it hard to know exactly which TE or which genomic copy is being expressed (65–67). This repetitive nature of TEs therefore poses a major challenge for resolving their expression using standard short-read RNA-sequencing approaches (srRNA-seq) (67). Novel computational frameworks, such as Telescope, SalmonTE, SQuIRE, TEtranscripts, and TEtools now enable locus-specific or family-level quantification of TE-derived transcripts (68, 69). While these tools are better suited for bulk RNA-seq data, they are less effective for single-cell analysis. To address this gap, scTE was specifically designed for single-cell RNA-seq data by He et al. (65). This tool allows researchers to integrate TE expression into single-cell transcriptome analyses (e.g., clustering, trajectory inference), revealing heterogeneity in TE activity across immune or developmental cell populations (65). Hunt et al. developed single-cell transposable element methylation sequencing (scTEM-seq), a cost-effective single-cell multi-omics method to assess DNA methylation at TEs using relatively low sequencing depth. By combining scTEM-seq with scRNA-seq, the method directly correlates TE methylation with TE transcriptional activation, revealing which cells upregulate TEs after hypomethylating treatment (70).

While highly useful, srRNA-seq is restricted by its limited read length (up to 300 bp in paired-end sequencing), which prevents the capture of distant exons within the same fragment and hinders accurate transcript reconstruction. In addition, it has difficulty mapping repetitive sequences such as TEs. In contrast, long-read sequencing methods (e.g., Nanopore sequencing and SMRT) sequence whole RNA or cDNA molecules, allowing direct reconstruction of full-length transcripts without the need for assembly (71).

Rigorous RNA-seq quality control (QC) is indispensable for TE expression studies because low-level genomic DNA carryover and unspliced pre-mRNA can artifactually inflate TE signal, particularly for the abundant intronic/intergenic and highly repetitive copies that dominate most TE families. Bulk RNA-seq libraries are especially susceptible: DNA-derived reads increase intergenic/intronic assignment, destabilize expectation–maximization (EM) procedures for resolving multimappers, and bias both locus- and family-level abundance estimates. Accordingly, QC should verify RNA integrity and library quality (e.g., RIN, rRNA content, duplication), document efficient DNase treatment, and interrogate mapping features indicative of DNA contamination and library failure, including elevated intergenic and intronic rates, reduced splice-junction usage and exon:intron ratios, abnormal 5′/3′ coverage profiles, unexpected insert-size distributions, incorrect strandedness, and excess multimapping (72–74). Enforcing these checks prior to TE quantification minimizes false positives and improves differential analysis robustness. Notably, the DNA-as-RNA artifact is largely a bulk RNA-seq problem. Most scRNA-seq platforms use oligo-dT priming and 3′/5′ tag capture, enriching for poly(A)+ molecules and thereby excluding non-polyadenylated genomic fragments; consequently, scRNA-seq predominantly measures polyadenylated TE transcripts (e.g., many LINE-1 and ERV RNAs) and is less prone to DNA-derived confounding, though ambient RNA and cell multiplets still warrant targeted QC (75, 76) For analysis, TE-aware quantifiers that model multimapping (e.g., Telescope for locus-level, TEtranscripts for family-level) should be preferred, and results interpreted in light of the known challenges of TE read assignment and polyadenylation heterogeneity (67, 68, 77).

However, beyond methodological progress, a critical question arises: what is the functional relevance of these epigenetic modifications? Emerging evidence suggests that dysregulated methylation and subsequent aberrant activity of TEs play an active role in shaping immune responses.

## 6 Transposable elements as regulatory agents in immune system modulation

**Figure 3:**
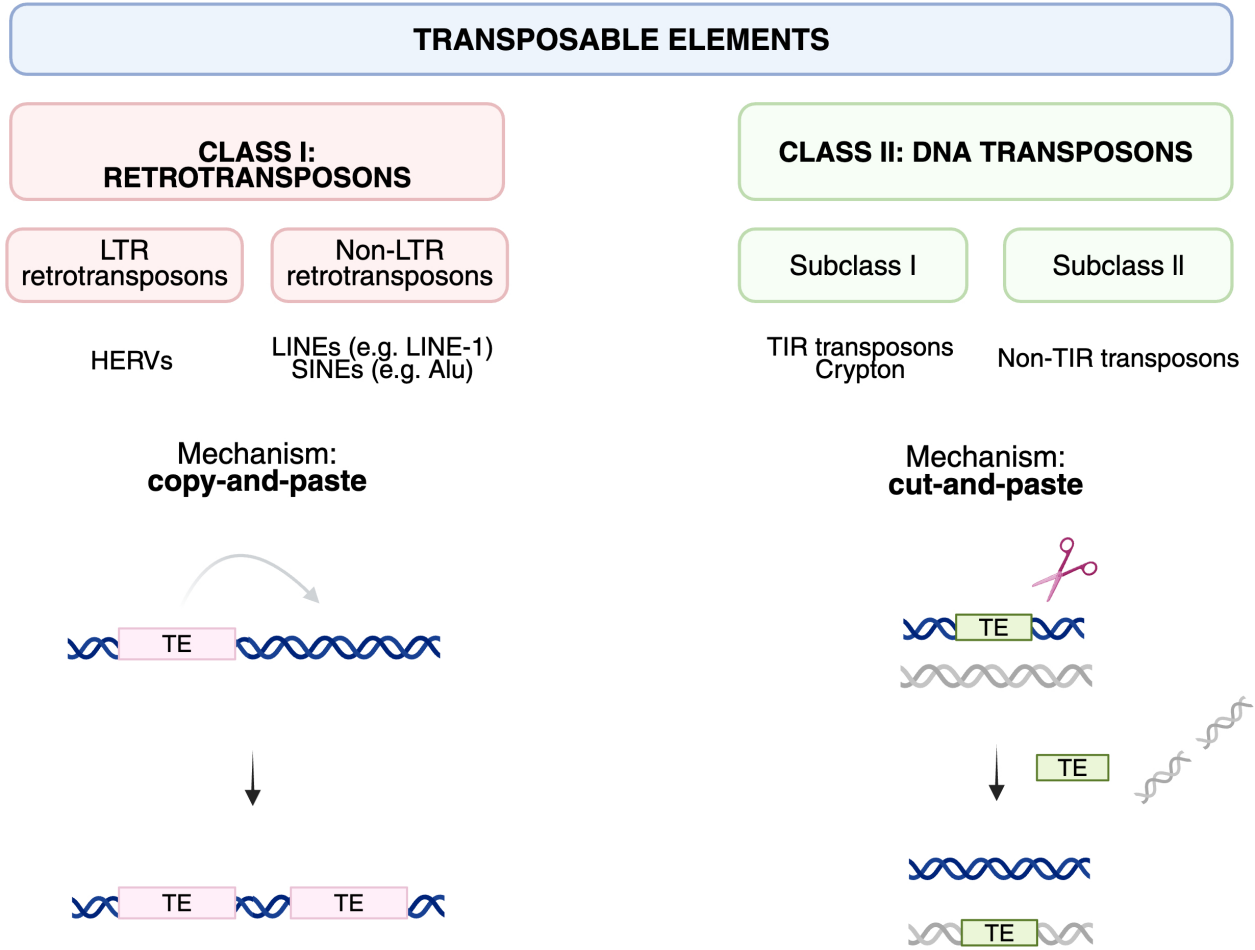
Major mechanisms by which transposable elements influence immunity.

Autoimmune diseases are a diverse group of disorders characterized by the immune system targeting the body’s own tissues and organs (78). This loss of self-tolerance results in inflammatory responses directed against self-antigens, leading to chronic inflammation and tissue damage (48). Through their mobility, transcriptional activity, and regulatory potential, TEs contribute to the evolution, modulation of immune responses, and sometimes dysregulation of immune responses (79, 80). TEs influence organisms through transposition, transcriptional activity and gene regulation, with both beneficial and harmful consequences (81). Below, we outline the major mechanisms by which TEs influence immunity (**Figure 3**).

Schematic overview of how transposable elements (TEs) shape immune function through their genomic mobility, transcriptional activity and regulatory potential. Left, *Transposition*: stress-induced “jumping” of TEs along the genome alters local gene architecture and can disrupt or activate nearby immune-related genes, generating genetic variation that may be either advantageous or pathogenic. Left bottom, *Regulatory functions of TEs*: inserted TEs can be exapted as cis-regulatory elements and act as enhancers, promoters or silencers, thereby rewiring gene expression programmes in immune cells. Right, *Transcriptional activity of TEs*: when epigenetic silencing (e.g. DNA methylation, H3K9me3) is relaxed, TE transcription produces RNAs and proteins that interface directly with the immune system. TE-derived peptides can be processed and presented as neoantigens, TE sequences can be exonized to create alternative splice isoforms, and TE-derived dsRNA or cytosolic DNA activate pattern-recognition receptors (RIG-I/MDA5–MAVS, cGAS–STING and TLRs), inducing type I interferons and pro-inflammatory cytokines in a “viral mimicry” response. Together, these processes link TE dysregulation to loss of immune tolerance and chronic inflammation in autoimmune diseases.

### 6.1 Transposition

The mobilization of TEs near protein-coding genes can generate new adaptive traits by reshaping the local regulatory landscape. Insertions of TEs into promoters or regulatory intronic regions can modify the expression of nearby genes and thereby influence phenotype (79). TEs can change genomic location through transposition or retrotransposition, and stress conditions such as infection or environmental challenges (e.g., temperature shifts, oxidative stress) are known to relax epigenetic repression and permit increased TE mobilization (82–84). This stress-associated mobility allows TEs to serve as sources of genetic and regulatory variation, introducing new insertions, modifying gene structure, and potentially activating or disrupting immune-related genes (31, 79, 81–84).

### 6.2 Transcriptional activity of transposable elements

#### 6.2.1 Neoantigen presentation

TE-derived peptides may be processed and presented by MHC molecules, acting as neoantigens (85). Consistent with this concept, Merlotti et al. recently demonstrated that non-canonical splice junctions between exons and TEs (“JETs”) in non-small cell lung cancer give rise to tumor-specific peptides that are naturally presented on HLA class I molecules and recognized by CD8+ T cells, establishing TE-derived sequences as a recurrent source of immunogenic neoantigens in patients (86). This can trigger T cell responses, which may be protective (e.g., in cancer), as T cells recognize TE-derived neoantigens on tumor cells, or pathological (e.g., in autoimmunity), as T cells mistakenly recognize TE-derived peptides on healthy cells (87, 88). The most comprehensive screening was performed by Saini et al. (2020), who used DNA barcode-labeled MHC-I multimers to detect CD8+ T cells recognizing 29 HERV-derived peptides from 18 HERV loci in hematological malignancy patients. The dominant families were HERVH-5, HERVW-1, and HERVE-3, with peptides restricted by HLA-A*02:01, HLA-A*01:01, HLA-B*07:02, and HLA-B*08:01 (89). HERV-specific T cells were present in 17/34 patients but less frequently in healthy donors.

#### 6.2.2 Alternative splicing and isoform diversity

TEs can influence alternative splicing by donating splice sites or regulatory elements, leading to the generation of immune protein isoforms with altered functionality, potentially reshaping immune signaling pathways (81, 90, 91). For example, Alu elements, the most abundant SINEs in the human genome, affect immune genes through several well-characterized mechanisms. The most compelling recent example involves CD58 (LFA-3), a critical T cell co-stimulatory molecule. A polymorphic Alu insertion within 100 bp of an alternatively used exon promotes skipping of CD58 exon 3, creating a frameshifted transcript and loss of function. RT-PCR validation confirmed this Alu variant acts as a functional splicing QTL (sQTL), and the variant is significantly associated with increased multiple sclerosis risk (92). Ilik et al. demonstrated that scaffold attachment factor B (SAFB) proteins bind adenosine-rich sequences within intronic LINE-1 and other transposon RNAs, preventing their recognition as exons and blocking the use of cryptic splice and polyadenylation sites. Loss of SAFB results in widespread TE exonization, activation of nearby cryptic splice sites, and premature transcript termination, effectively incorporating intronic TEs into transcripts. These findings establish TE exonization control as an active, sequence-guided host defense mechanism that safeguards transcriptome integrity while modulating alternative splicing landscapes (93).

#### 6.2.3 Innate immune sensing

When TEs are transcriptionally active, they produce RNAs and, in some cases, proteins that can be directly detected by innate immune sensors. Loss of epigenetic repression, such as DNA methylation or repressive histone marks, can lead to the transcriptional reactivation of TEs, particularly LINE-1 elements and ERVs (10, 94). This reactivation results in the accumulation of double-stranded RNA (dsRNA) and cytosolic DNA, which are potent activators of the innate immune system (23).

The innate immune system employs distinct pattern recognition receptor (PRR) systems to detect these TE-derived nucleic acids. The RIG-I/MDA5-MAVS pathway detects dsRNA formed through bidirectional transcription of TEs. L1 elements possess a bidirectional promoter in their 5′ UTR, comprising a sense promoter that produces canonical L1 mRNA (ORF1/ORF2) and an antisense promoter (L1−ASP) that transcribes into flanking host DNA. L1−ASP generates chimeric L1–gene transcripts (LCTs), some oriented antisense to host genes, which can form dsRNA that triggers innate immune sensing (95, 96). In addition to bidirectional transcription, endogenous dsRNA is also generated by IRAlus, formed when oppositely oriented Alu elements within the same transcript fold into long intramolecular dsRNA structures. These abundant Alu-derived duplexes are normally edited by ADAR1, which limits their immunogenicity, but accumulate as unedited dsRNA when RNA editing, splicing, or epigenetic TE silencing is disrupted (97, 98).

Upon dsRNA binding, these receptors signal through the mitochondrial adaptor protein MAVS (mitochondrial antiviral signaling protein) to activate interferon regulatory factors IRF3 and IRF7, as well as NF-κB, inducing type I interferons (IFN-α and IFN-β) and inflammatory cytokines (46, 47). Tunbak et al. demonstrated that depletion of the HUSH silencing complex leads to LINE-1-derived dsRNA production from bidirectional transcription of full-length hominid-specific L1 elements, with interferon induction dependent on both MDA5 and RIG-I, and importantly, LINE-1 overexpression from exogenous plasmid alone was sufficient to activate interferon signaling (38, 99, 100).

The cGAS-STING pathway serves as the primary sensor for cytoplasmic DNA. When LINE-1 elements are transcribed and undergo reverse transcription, the resulting cDNA and RNA-DNA hybrid intermediates accumulate in the cytoplasm where cyclic GMP-AMP synthase (cGAS) detects them (37, 40). cGAS catalyzes production of the second messenger 2’3’-cGAMP, which activates STING (stimulator of interferon genes), leading to TANK-binding kinase 1 (TBK1)-mediated phosphorylation of IRF3 and subsequent type I interferon production (41, 42). Additionally, Toll-like receptors (TLRs) provide a third layer of TE sensing: TLR3 in endosomes detects dsRNA, TLR7/8 recognize single-stranded RNA, and TLR9 detects unmethylated CpG DNA, which is a signature enriched in normally methylated TE sequences (48). This phenomenon, termed “viral mimicry,” describes how reactivated TEs produce molecular patterns resembling viral infection, thereby triggering antiviral defense pathways (47, 49–51). The term refers to the cell entering a pseudo-viral state due to TE reactivation rather than actual viral infection. Chronic activation of these pathways is a hallmark of several autoimmune diseases, such as type 1 diabetes (T1D), systemic lupus erythematosus (SLE), rheumatoid arthritis (RA), Sjögren’s syndrome, and systemic sclerosis (52, 101).

### 6.3 Regulatory functions of transposable elements

TEs frequently integrate into or near gene regulatory regions and can function as cis-regulatory elements that reshape immune gene expression (80). TEs may serve as enhancers, promoters or silencers (8). TEs frequently provide enhancer sequences that can activate nearby gene expression. These TE-derived enhancers are often tissue- or cell-type-specific and can rapidly introduce new regulatory elements into gene networks, especially during development and in certain cancers (18, 102). In some immune cells, TEs provide alternative promoter sequences that initiate gene transcription. This can result in alternative gene isoforms or non-canonical gene regulation, sometimes affecting gene expression far from the original TE insertion (102–104). Lastly, some TEs act as silencers, repressing gene expression either directly or by recruiting repressive chromatin modifiers (102). For instance, Buttler et al. identified a LINE-1–derived intronic enhancer (IFNAR1.L1M2a) within the IFNAR1 locus in human immune cells; CRISPR deletion of this TE element reduced basal IFNAR1 expression and abolished its up-regulation after type I interferon stimulation, dampening downstream interferon-stimulated gene responses, a pathway that is central to systemic lupus erythematosus and other autoimmune diseases (105). At a broader scale, Ye et al. showed that primate-specific endogenous retroviruses and Alu elements have seeded NF-κB and IRF1 binding motifs into human immune-cell enhancers, and that these TE-derived “inflammatory disease enhancers” are enriched for risk variants for lupus, RA, inflammatory bowel disease, psoriasis and related autoimmune traits (106).

## 7 Transposable elements in autoimmune diseases

Having a genetic predisposition alone is not sufficient to cause autoimmune disease, as shown by cases where identical twins, who share the same genetic makeup, do not both develop the condition (107, 108). This highlights the significant role of environmental factors in triggering disease onset (109).

Aberrant TE methylation and expression have been implicated in a broad range of autoimmune and immune-mediated diseases, including T1D, SLE, RA, multiple sclerosis, Sjögren’s syndrome, inflammatory bowel disease, among others (110–116).

We present below an overview of studies investigating TE methylation and expression in T1D, SLE and RA (**Table 1**).

**Table 1:** Overview of studies investigating transposable element methylation and expression in Type 1 diabetes, Systemic lupus erythematosus and Rheumatoid arthritis.

Disease	Reference	Sample/Cell type	Key findings	Clinical relevance
*Type 1 diabetes (T1D)*	Starskaia et al. (117)	CD4+, CD8+ T cells, CD4-CD8- lymphocyte fractions (preseroconversion)	Hypomethylation (*ARRDC2*, *PCBP3, TRAF3, IL32*), hypermethylation (*IRF5, DGKQ, TOX)*	Suggests causal role in early disease initiation
	Katsanou et al. (118, 119)	Peripheral blood (T1D group vs. healthy group)	Altered Alu (­ partial methylation mCuC+uCmC) and LINE-1 methylation patterns (­ total methylation, ¯ partial methylation uCmC)	Potential epigenetic biomarkers of T1D
	Tovo et al. (120)	PBMCs (new-onset T1D)	­ HERV H and HERV W *pol* expression	Implicates human endogenous retroviruses in b-cell damage
*Systemic lupus erythematosus (SLE)*	Sukapan et al. (113)	Neutrophils	LINE-1 hypomethylation, no changes in Alu, HERV-E and HERV-K	Hypomethylation independent of disease activity (SLEDAI score)
	Wu et al. (121)	CD4+ T cells	Hypomethylation of HERV-E LTR2C region	Links LTR methylation to HERV expression regulation
	Arteaga-Vasquez et al. (122)	Multiple leukocyte subsets	TE upregulation correlates with IFN-I activation; neutrophils show most differentially expressed TEs (LTR, ERV families)	TE overexpression sustains IFN responses; potential biomarker and therapeutic target in SLE
	Cutts et al. (123)	Sorted immune cells (CD4+ T, monocytes, B cells, NK cells)	>700 differentially expressed TEs	Correlates with ISG upregulation and clinical subtypes
*Rheumatoid arthritis (RA)*	Fu et al. (124) Brandt et al. (125) Bagni et al. (62)	Synovial fibroblasts, PBMCs	Abberant methylation (*IL6R, CAPN8, DPP4, DR3, CXCL12, TBX5*, and *IL-10)*	Disease-specific methylation signature
	Mucientes et al. (126)	PBMCs	Hypermethylation (*TBC1D22A, PRHOXNB*)	Disease-specific methylation signature
	Sohanforooshan Moghaddam et al. (127)	PBMCs	Hypomethylated PARP9 promoter; negative correlation with ESR/CRP	Potential marker of inflammation and activity
	Gosselt et al. (128)	Whole leukocytes	­ global methylation corresponds with poor MTX response	Predicts MTX non-response
	Ravaei et al. (129)	PBMCs	LINE-1 methylation interacts with RF/ACPA status	Personalized therapy stratification

### 7.1 Type 1 diabetes

T1D is an autoimmune disease where the body’s immune system destroys insulin-producing pancreatic cells, leading to insulin deficiency (130). There is strong evidence that neoantigens, when presented by pancreatic β-cells, can activate autoreactive T cells and contribute to the onset of T1D and potentially other autoimmune diseases (131). Recent research suggests that TEs may play a role in the development and regulation of T1D, particularly through their influence on gene expression and immune responses. TEs may act as a missing link between genetic predisposition and environmental triggers (e.g. viral infections) (112, 132). Recent longitudinal epigenomic analyses provide compelling evidence that DNA methylation changes precede the clinical onset of T1D, suggesting a potential involvement in disease initiation (117, 133–135). In a study profiling purified CD4+ T cells, CD8+ T cells, and CD4-CD8- lymphocyte fractions from children with increased genetic susceptibility, researchers obtained samples prior to the development of islet autoantibodies (117, 136). Findings included hypomethylation of the *ARRDC2* promoter in CD4+ T cells, hypomethylation of an intronic enhancer in *PCBP3* within CD8+ T cells, and hypermethylation of the *IRF5* promoter, a key regulator of the IFN-I pathway implicated in multiple autoimmune disorders. Other recurrently altered loci included *TRAF3* (hypomethylated in CD4+ and CD4-CD8- cells), *IL32* (hypomethylated promoter in CD8+ T cells with increased mRNA expression), *DGKQ* (hypermethylated in CD4+ and CD4-CD8- cells), and *TOX* (hypermethylated in CD4+ T cells). These genes have well-established roles in T cell activation, cytokine signaling, and immune tolerance, making them plausible mediators of early autoimmune activation (117).While not focused on TEs, these studies identified pre-clinical methylation changes in immune genes, suggesting epigenetic dysregulation precedes over autoimmunity. Katsanou et al. demonstrated that in the T1D group, the combined partial Alu methylation pattern (mCuC + uCmC) was significantly more frequent compared to healthy controls (41.9% vs. 36.0%, p = 0.004), whereas the prevalence of hypermethylated (mCmC) and hypomethylated (uCuC) Alu patterns did not differ between groups (118). The same research group subsequently performed a case-control study, where methylation of LINE-1 retrotransposons was quantified using combined bisulfite restriction analysis (COBRA). COBRA is a semi-quantitative method for assessing DNA methylation at specific CpG sites (119, 137). The total methylation rate (mC) of LINE-1 was higher in the T1D group compared to the control (median 47.3% vs. 46.5%, p = 0.005). Analysis of methylation patterns at two CpG sites within LINE-1 revealed a reduction in the partially methylated uCmC pattern in T1D patients (28.4% vs. 33.1%, p = 0.019), while the frequencies of the hypermethylated (mCmC), hypomethylated (uCuC), and alternative partial methylation (mCuC) patterns did not differ between groups (119). These findings appear paradoxical as individuals with T1D show *higher* total LINE-1 methylation (mC) but a *lower* frequency of the partially methylated uCmC pattern, while other patterns (mCmC, mCuC, uCuC) do not differ significantly. However, this is not necessarily contradictory. First, the increase in total mC is small, and even subtle, non-significant shifts toward fully methylated (mCmC) or alternative partially methylated (mCuC) states can raise the average methylation while uCmC declines. Second, total mC collapses all patterns into a single value and therefore obscures the *structure* of methylation across CpG dyads. Pattern-level analysis instead suggests a redistribution from mosaic uCmC states toward more uniformly methylated or unmethylated configurations. Finally, COBRA interrogates only two CpG sites in bulk blood, so these shifts may reflect a composite of modest locus-specific changes, altered leukocyte composition, and metabolic factors rather than a simple global hyper- or hypomethylation phenotype. Overall, the data support a subtle reorganization of LINE-1 methylation in T1D toward slightly higher average methylation but reduced uCmC heterogeneity rather than a straightforward global hypomethylation model (104). To assess HERV activity during the earliest stages of T1D, transcriptional levels of the *pol* genes from three major HERV families, HERV-H, HERV-K, and HERV-W, were measured in peripheral white blood cells of 37 children and adolescents with new-onset T1D and 50 age-matched healthy controls. The *pol* gene encodes the viral enzymes required for reverse transcription and integration, and its transcriptional activity serves as a marker of retroviral activation. Compared with controls, patients with T1D exhibited significantly higher expression of *HERV-H-pol* and *HERV-W-pol*. Specifically, *HERV-H-pol* transcripts were elevated by approximately 11% (p = 0.0001), while *HERV-W-pol* expression increased by about 15% (p < 0.0001) (120). The observed upregulation is particularly notable for *HERV-W*, as its envelope protein (HERV-W-env) has previously been shown to inhibit insulin secretion in pancreatic islets via TLR4 activation and to contribute to β-cell damage both directly and through immune-mediated mechanisms (138). In contrast, *HERV-K-pol* levels did not differ significantly between groups (120).

### 7.2 Systemic lupus erythematosus

SLE is a multisystemic autoimmune disease characterized by abnormal activation of the IFN-I pathway, which disrupts immune tolerance and results in the production of autoantibodies targeting components of the cell nucleus (52, 139). Early epigenetic studies demonstrated widespread DNA hypomethylation in T cells from patients with active SLE (57). This epigenetic instability extends to repetitive elements: analysis of interspersed repetitive sequences (IRSs) in neutrophils revealed significantly increased LINE-1 hypomethylation in SLE compared with healthy controls. LINE-1 methylation did not correlate with disease activity (SLEDAI score), indicating that this alteration is largely independent of disease severity (113). In contrast, methylation levels of Alu, HERV-E, and HERV-K were not significantly different between groups, supporting the concept that LINE-1 hypomethylation represents a relatively stable disease-associated epigenetic feature rather than a dynamic marker of flare intensity, and raising the possibility that LINE-1 depression contributes to SLE pathogenesis (113). In a separate study researchers focused on the HERV-E LTR2C region, which contains three key CpG sites that can be methylated. Bisulfite sequencing analysis of CD4+ T cells from SLE patients revealed hypomethylation of this region compared to controls. These results suggest that lower methylation of the LTR2C region may lead to higher HERV-E expression, indicating that LTR methylation regulates HERV activity in lupus CD4+ T cells (121).

Transcriptomic studies now place TE dysregulation directly upstream of interferon pathway activation. Arteaga-Vazquez et al. reanalyzed RNA-seq data from multiple leukocyte subsets in SLE and showed that a substantial proportion of patients display an IFN-I–high gene expression profile marked by elevated IFNα/IFNβ and ISGs, and investigated associations among TE expression, interferon responses, and clinical disease activity (122). Importantly, individuals who later developed SLE displayed mildly elevated circulating IFN-I and IFN-γ levels together with autoantibodies before clinical diagnosis, and IFN-positive patients showed TE upregulation across all leukocyte subsets, with neutrophils harboring the greatest number of differentially expressed TEs. Upregulated elements were predominantly derived from LTR/ERV families, with additional contributions from LINEs, SINEs, and DNA transposons (122, 140, 141). A related analysis across six leukocyte types similarly linked distinct interferon response patterns to increased TE expression, again identifying neutrophils as the most prominently affected compartment (142). In a larger cohort, a TE-high subgroup of SLE patients was defined by widespread LTR retrotransposon activation, strong IFN signaling, and altered immune cell composition characterized by neutrophilia and reduced regulatory T cells, with neutrophils representing the principal cellular source of TE overexpression (143). Complementary locus-specific RNA-seq of sorted immune populations identified more than 700 differentially expressed TEs, largely cell-type restricted and associated with distinct clinical subphenotypes; TE expression strongly correlated with interferon-stimulated genes (ISGs) such as *ISG15*, *LY6E*, *IRF7*, and *BST2* and with antiviral and inflammatory pathways in a cell-type–dependent manner (123). Together, these findings support a model in which aberrant TE overexpression — particularly in neutrophils — generates endogenous immunostimulatory nucleic acids that engage innate nucleic acid sensors and perpetuate the IFN-I signature in SLE through viral mimicry–like mechanisms (122, 143).

### 7.3 Rheumatoid arthritis

RA is a common autoimmune disease that mainly affects the joints but can also involve other organs and systems, such as the lungs, blood vessels, and skin (144). RA develops through a combination of genetic, environmental, and epigenetic factors, with changes in DNA methylation, histone acetylation, and microRNAs influencing immune cells and synovial fibroblasts, contributing to disease pathogenesis (114, 144, 145). Synovial fibroblasts are central players in RA pathogenesis, contributing to chronic inflammation and joint destruction. DNA methylation profiling of RA synovial fibroblasts and peripheral blood mononuclear cells (PBMCs) identified aberrant methylation in several genes (e.g., *IL6R, CAPN8, DPP4, DR3, CXCL12, TBX5*, and *IL-10*) (56, 62, 124, 125). A recent study provides compelling evidence that RA is characterized by a specific DNA methylation signature that distinguishes patients from healthy controls. Among the most notable findings was the hypermethylation of CpG sites in *TBC1D22A* (cg08161306, 19-Down-cg08161306) and *PRHOXNB* (cg21950155) in RA patients (126). Another study demonstrated significant hypomethylation of the *PARP9* promoter in PBMCs compared to healthy controls (p<0,001). Moreover, PARP9 promoter hypomethylation was negatively correlated with inflammatory markers erythrocyte sedimentation rate (ESR), an indirect measure of the level of inflammation, and C-reactive protein (CRP), a direct measure of inflammation, suggesting a link between lower methylation levels and higher disease activity (127, 146). In a prospective study of early RA patients, Gosselt et al. reported that higher baseline global leukocyte DNA methylation was associated with poor response to methotrexate (MTX), the first-line disease-modifying antirheumatic drug (147) after three months of therapy. Using both liquid chromatography-tandem mass spectrometry and LINE-1 methylation analysis, they demonstrated that patients with elevated methylation levels at baseline exhibited a smaller reduction in disease activity and were more likely to be classified as non-responders according to EULAR criteria (128). In a separate study, Ravaei et al. examined whether LINE-1 methylation patterns in PBMCs could predict responsiveness to MTX in early RA. As opposed to previous study, they reported no significant difference in LINE-1 methylation levels between non-responders, moderate responders, and good responders to MTX. Notably, in patients double positive for rheumatoid factor (RF) and anti-citrullinated protein antibodies (ACPA), higher LINE-1 methylation levels were significantly associated with a better MTX response. In seropositive RA, which is marked by strong immune-complex–driven inflammation, higher LINE-1 methylation may suppress retroelement-related nucleic acid signals and reduce innate immune activation, creating an inflammatory environment that responds better to MTX’s immunomodulatory effects. In contrast, among seronegative patients (RF–/ACPA–), lower LINE-1 methylation levels correlated with more favorable outcomes. Patients with single antibody positivity (RF+ or ACPA+ alone) showed no clear methylation-response relationship (129). This study provides the first evidence that integrating LINE-1 methylation with RF and ACPA status could improve prediction of MTX responsiveness in early RA. Such an approach may contribute to more personalized treatment strategies, allowing early identification of patients likely to benefit from MTX and sparing others from ineffective therapy.

## 8 Environmental induction of HERV activation

### 8.1 Exogenous infections

While aberrant TE methylation and expression clearly contribute to immune dysregulation in T1D, SLE, and RA, genetic predisposition and intrinsic epigenetic alterations alone do not fully account for disease onset, a broad range of environmental stimuli have been shown to directly trigger HERV and TE activation, providing the exogenous component that bridges susceptibility and overt autoimmunity.

#### 8.1.1 Viral

Exogenous viral infections are strongly associated with the transactivation of HERVs, representing a key mechanism linking environmental exposure to TEs dysregulation. A wide range of DNA and RNA viruses - including Epstein-Barr virus, herpesvirus, retrovirus, and respiratory viruses - have been shown to induce HERV expression across multiple cell types. First, viral proteins can directly transactivate HERV loci by interacting with their LTRs via host transcription factors such as NF- κB, AP1, and Sp1 (148). Second, viral infection induces intracellular signaling cascades (e.g., PKC and interferon pathways) that increase chromatin accessibility and promote TEs transcription (149). Third, viruses can disrupt epigenetic silencing by modulating key regulators such as DNA methyltransferases and histone modifiers, leading to loss of repressive marks (e.g., H3K9me3) and derepression of HERV loci (148). Functionally, virus-induced HERV activation contributes to autoimmunity through several mechanisms, including molecular mimicry, activation of innate immune sensors by HERV-derived nucleic acids, and amplification of interferon-driven inflammation. These processes establish a mechanistic link between viral infection, HERV activation, and the breakdown of immune tolerance, positioning HERVs as key mediators of the genetic–environmental interface in autoimmune diseases such as SLE (150).

In the context of SLE, viral infections contribute to HERV activation primarily through epigenetic mechanisms. External triggers, including infections, induce global DNA hypomethylation and chromatin remodeling, leading to derepression of HERV loci and increased transcription of interferon-responsive genes (150).

#### 8.1.2 Bacterial

In addition to viral triggers, emerging evidence suggests that bacterial infections may also contribute to TE activation. For example, infection with *Mycobacterium avium* subspecies *paratuberculosis* has been proposed as a potential environmental risk factor for T1D and has been associated with the reactivation of HERV-W expression (151). Once activated, HERVs can amplify immune responses through innate immune receptor engagement and cytokine production, contributing to β-cell destruction in T1D, interferon-driven autoimmunity in SLE, and chronic synovial inflammation in RA (152). Although the precise molecular mechanisms remain less well defined than for viral infections, bacterial-induced inflammation and immune activation are thought to promote epigenetic dysregulation, thereby facilitating HERV transcription and contributing to autoimmune pathogenesis.

### 8.2 Inflammation

Oxidative stress is increasingly recognized as a key signal that can reactivate normally silenced HERVs. This occurs mainly through redox-driven epigenetic changes and inflammatory signaling, with strongest evidence in neuroinflammatory disease and cancer. In multiple sclerosis, oxidative stress promotes DNA hypomethylation at HERV loci by oxidizing guanine (8-oxoG) and converting cytosine to 5-hydroxymethylcytosine, blocking DNA methyltransferase binding and favoring HERV transcription (153). Oxidative stress also inhibits histone deacetylases, increasing histone acetylation and opening chromatin around HERV promoters (153). Reviews on HERVs and inflammation describe a tight association between chronic oxidative stress, inflammation, and HERV upregulation, proposing a feed-forward loop where each amplifies the other (154).

### 8.3 Chemical and physical factors

#### 8.3.1 Radiation

UVB radiation is a potent environmental trigger of HERV activation, linking physical exposure to TE dysregulation through epigenetic remodeling and antiviral signaling pathways. Increasing evidence indicates that UVB-induced HERV expression contributes to both oncogenic transformation and autoimmune disease. In melanoma, UVB stimulates the expression of HERV-K pol and env genes, leading to the production of retroviral particles that may fuel tumor progression (155). In SLE, UVB exposure decreases HERV-E LTR2C methylation and upregulates HERV-E mRNA expression in CD4+ T cells (121). In keratinocytes, UVB-induced activation of HERVs leads to accumulation of dsRNA, which activates the innate antiviral RIG-I/MDA5/IRF7 pathway, inducing IFN-I and interferon-stimulated genes, and ultimately promoting apoptosis and reduced cell proliferation, which may contribute to skin inflammation and skin lesions in SLE/DLE (156). Recent research indicates that HERV-derived dsRNAs, resulting from DNA hypomethylation, contribute to SLE pathogenesis by activating the RIG-I/IRF3 pathway, which promotes pathogenic Th1 differentiation via the IFN-I/STAT1 axis while inhibiting regulatory T cell (Treg) development through SMAD3 signaling (157). Together, these findings highlight a UVB - HERV axis in which radiation-induced epigenetic changes and antiviral immune responses converge, linking environmental exposure to both oncogenic transformation and immune dysregulation.

#### 8.3.2 Drugs and toxins

Antipsychotic drugs have been linked to the activation of HERVs transcription. For example, valproic acid induces dose-dependent upregulation of several HERV families, including HERV-W, in human brain cell lines (158). Additionally, the pesticide dieldrin has been reported to activate HERV-driven enhancers in human T cells (159), suggesting that both pharmaceutical and environmental agents can influence HERVs activity.

## 9 Conclusion and future prospects

In this review, we summarized accumulating evidence that TEs are not merely genomic passengers but active participants in immune regulation and, potentially, autoimmune disease pathogenesis. TEs occupy almost half of the human genome and are among the most heavily methylated regions, reflecting strong evolutionary pressure to maintain them in a repressed state (8, 21, 31). Loss or reshaping of this epigenetic control has two principal consequences that are directly relevant to autoimmunity: (i) reactivation of TE transcription, with production of immunostimulatory nucleic acids and peptides that can engage innate and adaptive immunity; and (ii) rewiring of gene regulatory networks, as TE-derived regulatory elements modulate expression of immune genes and cytokine pathways (51, 80, 81). Together, these processes provide a plausible mechanistic link between genetic predisposition, environmental triggers and chronic immune activation in autoimmune disorders.

Across the diseases reviewed, TE methylation patterns more often resemble disease-associated traits than rapidly fluctuating state markers. In SLE, LINE-1 hypomethylation in neutrophils did not correlate with SLEDAI, and no significant differences were observed for Alu, HERV-E or HERV-K, suggesting a relatively stable, disease-associated hypomethylation phenotype rather than a dynamic marker of flare severity (113). A similar picture emerges from longitudinal T1D cohorts: DNA methylation changes in immune genes precede seroconversion and clinical onset, supporting a role in early disease initiation rather than reflecting established inflammation (117, 133). In RA, LINE-1 methylation has shown limited value as a simple activity marker, but in combination with serostatus, it may help stratify MTX response, again pointing towards trait-like, pathway-level information rather than direct correlation with short-term inflammatory burden (128, 129). These observations are important conceptually. They imply that TE methylation is more likely to capture underlying susceptibility architecture shaped by genetics, early-life exposures and long-standing immune dysregulation, than transient changes in disease activity. This does not preclude dynamic modulation by flares or therapy, but it argues that TE methylation should be interpreted as a relatively stable layer of epigenetic risk that interacts with environmental triggers, rather than as a straightforward biomarker of current disease severity (33, 62, 109).

Historically, autoimmune epigenetics has been framed in terms of “global hypomethylation”, particularly in SLE T cells (57). The TE-focused literature refines this view in two ways. First, hypomethylation is family- and locus-specific rather than uniform: in SLE, LINE-1 elements in neutrophils and HERV-E LTR2C in CD4+ T cells are altered, while other repetitive families remain unchanged (113, 121). In T1D, modest but statistically significant shifts in Alu and LINE-1 COBRA patterns suggest a redistribution among partially and fully methylated states rather than a simple genome-wide loss of methylation (118, 119). Second, recent RNA-seq–based studies show that TE overexpression is highly cell-type specific, with distinct TE families dominating in different leukocyte subsets and associating with defined clinical sub-phenotypes (122, 123, 143). These findings argue against the idea of a uniform, global TE hypomethylation signature in autoimmunity. Instead, they support a model in which specific TE families in defined cell types become dysregulated and participate in disease-relevant pathways: LINE-1 and LTR/ERV families in neutrophils and T cells in SLE, selected HERV families in T1D, and LINE-1 methylation interacting with autoantibody status in RA (113, 120, 129, 132, 143). Methodologically, this underscores the limitations of surrogate “global” measures such as bulk LINE-1 COBRA or pyrosequencing, which average over thousands of loci and cell types and may obscure disease-critical, locus-specific changes. The “viral mimicry” model provides a compelling mechanistic framework: TE hypomethylation and loss of repressive histone marks permit bidirectional transcription of ERVs and LINE-1, generating dsRNA and cytosolic DNA that activate RIG-I/MDA5–MAVS, cGAS–STING and TLR pathways, driving type I interferon responses (35, 36, 40, 41, 46, 47, 49–51, 160). Experimental work with DNMT inhibitors in cancer and genetic manipulation of TE repression complexes has demonstrated that forced TE reactivation is sufficient to trigger interferon-stimulated gene (ISG) upregulation and antiviral programmes (38, 49, 50). In SLE, multiple lines of evidence now converge on this model. Arteaga-Vazquez et al. and Wang et al. identified TE-high SLE patient subgroups with widespread LTR/ERV upregulation, strong correlations between TE expression and IFN signatures, and enrichment of nucleic acid sensor pathways, with neutrophils emerging as a key TE-expressing compartment (122, 142, 143). Cutts et al. further showed that TE overexpression in specific cell types co-segregates with ISG expression and clinical subphenotypes (123). These data strongly support TE-driven viral mimicry as a plausible contributor to the sustained IFN-I signature in SLE. However, strict causality in human autoimmunity remains to be definitively proven. TE activation could be both driver and passenger: chronic IFN exposure and inflammatory signalling can themselves alter chromatin, upregulate TEs and reinforce a positive feedback loop (47, 52). Disentangling cause and effect will require longitudinal sampling, functional perturbation studies (e.g., pharmacological or genetic suppression of specific TE families or their reverse transcriptase) and integration with genetic risk variants in nucleic acid sensing pathways (e.g., IFIH1, TREX1, cGAS-STING components) (40, 41, 140).

Beyond their role as innate immune agonists, TEs are extensively exapted as cis-regulatory elements at immune genes (8, 80, 102, 103). TE-derived enhancers and promoters have seeded binding motifs for transcription factors such as NF-κB, IRF1 and STATs in many immune cell types, and these TE-originated regulatory elements are enriched for autoimmune risk variants (2, 102, 103). Recent work showing a LINE-1–derived intronic enhancer within the IFNAR1 locus that is required for appropriate IFNAR1 induction after type I interferon stimulation provides a concrete example of TE-mediated fine-tuning of antiviral and autoimmune pathways (105). At the same time, TE exonization can generate alternative isoforms of interferon pathway components, further diversifying immune responses (90, 91). From an autoimmune perspective, this means that altered TE methylation can have indirect regulatory consequences, even in the absence of overt TE overexpression or viral mimicry. Hypomethylation of TE-derived enhancers or promoters may change chromatin accessibility and transcription factor occupancy at nearby genes, subtly shifting thresholds for T cell activation, cytokine production or interferon responsiveness. Integrating TE methylation maps with eQTL, sQTL and chromatin interaction data in disease-relevant immune cells will be crucial to identify which TE insertions act as functional regulatory hubs in autoimmunity. The current literature is shaped by important methodological constraints. Many early studies relied on bulk surrogate assays (LINE-1/Alu COBRA or pyrosequencing) in heterogeneous blood cell populations, which cannot distinguish locus-specific or cell-type-specific changes and are vulnerable to confounding by shifts in leukocyte composition or treatment (31, 60, 118, 119, 137). Bisulfite-based approaches remain a gold standard but are limited in repetitive regions and by DNA degradation (13, 58–60). Recent technical advances offer powerful solutions. Long-read sequencing (ONT, SMRT) allows direct, base-level methylation detection across full-length TEs and precise mapping of individual insertions, enabling truly locus-resolved TE epigenomics (63, 64, 71). Dedicated TE expression pipelines (Telescope, TEtranscripts, scTE) and single-cell multi-omic methods such as scTEM-seq now make it possible to relate TE methylation and transcription within individual immune cells (65, 67–70). At the same time, TE-aware RNA-seq analysis requires stringent QC to exclude DNA contamination and properly model multimapping reads. Failure to do so risks overestimating TE expression and misinterpreting artefacts as biology (67, 72–74). Interpretation is further complicated by reverse causality and confounding. Disease activity, cytokine milieu, infections, medications (including DNMT and HDAC inhibitors) and comorbidities can all affect TE methylation and expression (33, 47, 51, 62, 84). The field therefore needs carefully designed longitudinal and interventional studies with detailed clinical, treatment and environmental annotation, ideally including pre-disease samples where available, as in T1D and preclinical SLE cohorts (117, 133–136, 140, 141).

Despite these challenges, several clinically relevant themes are emerging. First, TE methylation and expression signatures have biomarker potential. In T1D, altered Alu and LINE-1 methylation patterns and HERV-H/W upregulation in new-onset patients suggest avenues for early diagnosis or risk stratification (118–120, 137). In SLE, TE-high interferon-positive subgroups might define distinct molecular endotypes with prognostic or therapeutic relevance (122, 123, 142, 143). In RA, combining LINE-1 methylation with RF/ACPA status improved prediction of MTX response, pointing to epigenetic markers as components of precision medicine strategies (128, 129). Second, TEs represent therapeutic double-edged swords. In oncology, deliberate TE reactivation with hypomethylating agents is being exploited to induce viral mimicry and enhance anti-tumor immunity (49, 50). In individuals predisposed to autoimmunity, similar epigenetic interventions could theoretically exacerbate or unmask autoimmune disease by amplifying endogenous nucleic acid signaling. Conversely, targeted suppression of pathogenic TE families, through reverse transcriptase inhibitors, antisense oligonucleotides, or CRISPR-based epigenetic editing, might dampen IFN-driven pathology in disorders such as SLE or Aicardi-Goutières syndrome (40, 43, 51). these possibilities remain largely speculative in the autoimmune context and require rigorous preclinical safety and efficacy studies. Finally, TE methylation patterns may capture aspects of the gene–environment interface that are not accessible by genetics alone. Environmental drivers classically implicated in autoimmunity, for example, viral infections, smoking, ultraviolet radiation, diet and microbiome shifts, are all capable of perturbing TE regulation (82–84, 101, 148, 152, 154, 155). Mapping how such exposures reshape TE methylation and expression in genetically susceptible individuals may help explain why only a subset of genetically at-risk people develop overt disease and could identify novel windows for prevention.

TEs are emerging as more than passive genomic relics, they are densely methylated, epigenetically dynamic elements that can act as endogenous sources of immunostimulatory nucleic acids, neoantigens and regulatory sequences at immune loci. In T1D, SLE and RA, available data collectively support a model in which selective dysregulation of TE methylation and expression contributes to the breakdown of immune tolerance, sustains interferon-driven inflammation and modulates responses to therapy. Rather than a uniform global hypomethylation phenomenon, autoimmunity appears to involve disease- and cell-type-specific TE signatures, particularly affecting LINE-1 and LTR/ERV families in defined leukocyte subsets. At the same time, the field is still in an early, association-dominated phase. Most studies are small, cross-sectional, and use surrogate measures that cannot resolve locus- or cell-specific effects. Causality, especially for TE-driven viral mimicry in human autoimmune disease, remains strongly suggested but not yet definitively demonstrated.

In summary, TEs provide a mechanistic framework linking genetic susceptibility, epigenetic regulation, and environmental triggers in autoimmune disease. Clarifying when TE dysregulation is a driver, a modifier, or a consequence of immune pathology will be essential. With the rapid maturation of long-read sequencing, single-cell epigenomics and integrative computational methods, research on TE methylation in autoimmunity is poised to move from association to mechanism, with the potential to yield new biomarkers and therapeutic avenues that could ultimately improve outcomes for patients with autoimmune disorders.
